# Enhanced Mechanical and Dielectric Properties of Polyurethane Elastomers Containing Modified SiO_2_

**DOI:** 10.1021/acsomega.4c08565

**Published:** 2024-11-15

**Authors:** Miaomiao Qian, Xinru Wang, LiYang Yao, Yanchao Zhu

**Affiliations:** College of Chemistry, Jilin University, Changchun 130012, China

## Abstract

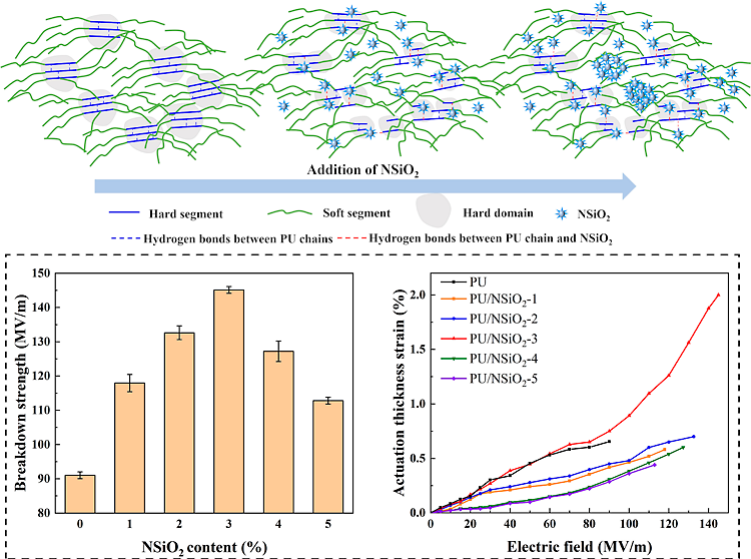

A novel and straightforward approach was employed to augment the dielectric constant and diminish the elastic modulus of polyurethane (PU) through the integration of modified silica (NSiO_2_) into the matrix. The incorporation of NSiO_2_ resulted in the disruption of N–H/C=O hydrogen bonds between the PU chains, which subsequently led to an enhancement in the polarizability of the chains and an increase in the dielectric constant of PU. Concurrently, the addition of NSiO_2_ resulted in a reduction of the elastic modulus (*Y*) of PU, which was attributed to the disruption of the hard domains of PU. The concurrent increase in the dielectric constant and reduction in *Y* give rise to a 60% enhancement in electromechanical sensitivity at 10^3^ Hz. Furthermore, PU/NSiO_2_ displays robust electrical breakdown strength due to the insulation properties of SiO_2_. At a NSiO_2_ content of 3%, the breakdown strength of PU/NSiO_2_-3 increases to 145.12 MV/m, which is 1.6 times that of PU (91.03 MV/m). This study presents a novel approach to the design and preparation of PU with a high dielectric constant and enhanced electrical breakdown strength.

## 1 Introduction

Dielectric elastomers (DEs) represent a novel and promising class of electroactive polymer materials that have garnered significant interest due to their numerous advantageous properties, including rapid response, high energy density, lightweight construction, high coupling efficiency, large strain, and flexibility.^1−3^ These diverse properties offer promising applications in actuators,^4^ soft robotics,^5^ artificial muscles,^6^ and energy harvesters.^7^ A number of materials have been identified as potential DEs, including acrylic elastomers,^8^ silicone rubbers,^9^ polyurethanes (PUs),^10,11^ and natural rubber.^12^ Among these materials, PU is a promising DE due to its high dielectric constant (ε_r_), recyclability, fast electric field response, and high energy density.^13,14^ However, the limitations of PU as a DE include its high elastic modulus (*Y*), low breakdown strength, and requirement of larger field strengths to obtain a larger deformation. One common method to increase the deformation is to add metal particles^15^ or inorganic conductive particles^16^ such as carbon nanotubes and graphene nanoplatelets. A distinct disadvantage of introducing these conductive particles into the elastomer matrix is that they usually decrease breakdown strength.

It is well established that PU is constituted of soft and hard segments (SS and HS), with the HS being formed by a reaction between a diisocyanate and a low-molecular-weight diol and the SS comprising polyols.^17^ The thermodynamic incompatibility of the HS and SS results in a unique microphase-separated structure. Subsequently, the HS and SS will undergo crystallization, resulting in the formation of hard and soft domains (HD and SD),^18^ respectively. The HD represents the physical cross-linking point of PU, which affects the mechanical properties of PU.^19^ Furthermore, a considerable number of N–H/C=O hydrogen bonds are present in the HS of PU, which constrains the mobility of the polarized groups and the dipole polarizability of PU.^20^ Accordingly, the disruption of the HD and hydrogen bonds can result in a reduction in the mechanical properties and an increase in the polarizability of PU, which will lead to a decline in the elastic modulus and an improvement in the ε_r_ of PU. For that purpose, some research has been conducted with the objective of enhancing the dielectric properties through the disruption of N–H/C=O hydrogen bonds in PU.^21−23^ However, a substantial alteration within the PU network resulted in a notable decline in mechanical strength, accompanied by a considerable reduction in the breakdown field. In general, it is necessary to establish a rational balance between the mechanical and dielectric properties, which is of substantial importance in the practical application of the DE.

Silica (SiO_2_) is a highly versatile reinforcing filler that can greatly enhance the mechanical properties of elastomeric composites.^24^ Due to its excellent insulating properties, SiO_2_ is widely used in DE to improve their electric breakdown strength.^25,26^ The combination of SiO_2_’s high strength and light mass makes it a viable option for disrupting the HD of PU, which in turn reduces the elastic modulus. However, the presence of a large amount of hydroxyl groups on the surface of SiO_2_ results in poor compatibility with the polymer matrix. A variety of techniques have been employed to facilitate the dispersion of SiO_2_ and enhance the interaction between SiO_2_ and the matrix, including surface modification of SiO_2_, the addition of a coupling agent, and the implementation of novel processing methods.^27−29^ For example, [3-(triethoxysilyl) propyl] tetrasulfide has been employed as a coupling agent for SiO_2_-filled natural rubber compounds to enhance the interfacial interaction between SiO_2_ and natural rubber.^30^ Wu et al.^31^ employed 3-aminopropyltriethoxysilane (APTES) to modify SiO_2_; the modified SiO_2_ exhibited uniform dispersion in PU and enhanced compatibility with the matrix. Amirhossein et al.^32^ found that the amine functional group in APTES could alter the interactions and electronic features at the silica–polymer interface.

In light of these findings, the objective of this study was to synthesize APTES-modified SiO_2_ (NSiO_2_) through chemical grafting and integrate it with PU to produce PU/NSiO_2_ composites. The effect of NSiO_2_ content on mechanical and dielectric characteristics was investigated. The incorporation of NSiO_2_ may cause the HD of PU to break up into multiple small hard domains and form a kind of nonideal two-phase system with a fuzzy interface, which could appropriately reduce the elastic modulus. The amino (N–H) group of NSiO_2_ has the potential to disrupt the hydrogen bonds between the PU chains, thereby increasing the dipole polarization of the PU matrix and consequently enhancing the ε_r_ of PU. Furthermore, the electrical breakdown strength of PU is adjusted to 145.12 MV/m by controlling the NSiO_2_ content. The objective is to present a straightforward approach to the design and preparation of soft materials with a high dielectric constant and good electrical breakdown strength.

## 2 Experimental Section

### 2.1 Materials

3-Aminopropyltriethoxysilane (APTES, 98%) and polypropylene glycol (PPG) with a molecular weight of 1000 and 2000 g/mol were procured from Aladdin (China). Tolylene-2,4-diisocyanate (TDI, 98% purity) was procured from Macklin (China). Dimethylolpropionic acid (DMPA), dibutyltin dilaurate (DBTDL), and triethylamine (TEA) were procured from Sinopharm Chemical Reagent Co., Ltd. (Shanghai, China). The solvent, acetone, was supplied by XiLONG Scientific, while absolute ethanol was provided by TianTai Chemical Reagent Co., Ltd. (Tianjin, China). Commercially available silica was procured from Shanghai Kaiyin Chemical Co., Ltd. The product is manufactured by Shanghai LK Chemical Industry Co., Ltd. and is marketed under the brand name LK-325. The laboratory purified deionized water.

### 2.2 Sample Preparation

#### 2.2.1 Synthesis of NSiO_2_ Nanoparticles

**Figure 1:**
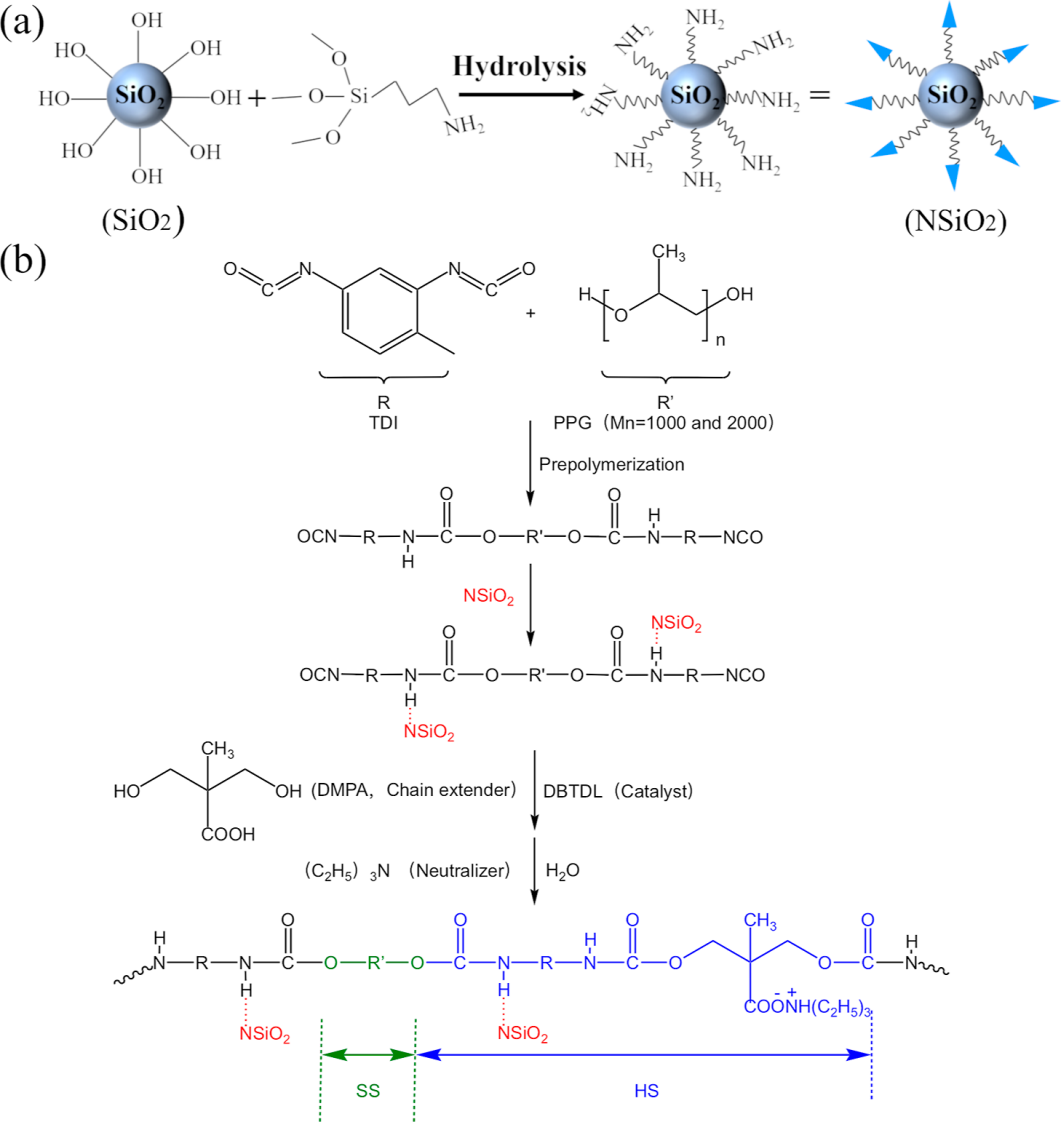
Schematic representation of preparation of NSiO_2_ and PU/NSiO_2_ composites.

As recommended in the literature, SiO_2_ was modified with APTES to introduce NH_2_ groups (Figure 1).^33^ First, silica was added to ethanol. Following ultrasonication for 0.5 h, a silica ethanol dispersion was obtained. Subsequently, the APTES ethanol dispersion was added dropwise to the SiO_2_ aqueous dispersion, maintaining a SiO_2_ to APTES mass ratio of 8.3:1. The resulting mixture was then heated to 80 °C for 3 h. After this, the mixture was centrifuged three times in ethanol and subsequently dried in a vacuum oven for 24 h, thus yielding NSiO_2_ nanoparticles.

#### 2.2.2 Synthesis of PU/NSiO_2_ Composites

The synthesis of PU/NSiO_2_ was conducted following the formulation shown in Table S2, as illustrated in Figure 1b. PPG_1000_ and PPG_2000_ (with a 9:1 mol ratio) and TDI (with a 2:5 mol ratio of OH to NCO) were reacted in a four-necked flask with a stirring device under a nitrogen atmosphere at 70 °C for 1.5 h to form an isocyanate-terminated prepolymer. Subsequently, the NSiO_2_ solution in acetone was introduced into the four-necked flask, and the reaction was continued at 70 °C for 0.5 h. DMPA was introduced as a chain extender, DBTDL was added as a catalyst, and acetone was subsequently introduced into the flask in an orderly fashion. The reaction mixture was then allowed to react at 60 °C for 2 h. Subsequently, TEA was added as a neutralizer. Following a 10 min neutralization reaction, the mixture was emulsified by the addition of distilled water and stirred at 2000 rpm for 1 h. PU/NSiO_2_ composite films were prepared by pouring the mixed aqueous dispersion into a polypropylene disk at 60 °C for 20 h in a vacuum oven. For comparison, PU and PU/SiO_2_ samples were also prepared by the same synthesis method. The PU/SiO_2_ and PU/NSiO_2_ composites with varying filler content were designated as indicated in Table S1.

### 2.3 Characterization

Fourier transform infrared spectroscopy (FTIR, Nicolet is5, Bruker, Saarbrücken, Germany) was employed to conduct FTIR spectroscopy of fillers. The FTIR spectra of PU/NSiO_2_ composites were obtained via Fourier transform infrared spectroscopy (Bruker V70, Bruker Co.) using attenuated total reflection (ATR) mode (Golden Gate single reflection diamond ATR, Specac.com) with 32 scans and a resolution of 2 cm^–1^.

The morphology of the fillers and the cross-sectional microstructure of PU/NSiO_2_ were observed using a field-emission scanning electron microscope (SU8020, Hitachi, Tokyo, Japan). The cross-sectional microstructure of PU/NSiO_2_ was obtained by brittle fracture of samples using liquid nitrogen.

A thermogravimetric analysis was conducted by using a NETZSCH STA449F3 differential thermal analyzer. The temperature was increased from room temperature to 800 °C at a rate of 10 °C/min under nitrogen atmosphere conditions.

The dynamic mechanical properties of PU/NSiO_2_ were evaluated using a DMA850 instrument (TA Corp., USA) at a 0.1% strain and a 1 Hz frequency. The samples were heated at a rate of 3 °C/min from a temperature of −50 to 80 °C.

Tensile tests of PU/NSiO_2_ were conducted by using a universal testing machine (CMT-20) with a stretching rate of 150 mm/min. The tensile specimens were rectangular in shape, with dimensions of 60 mm in length, 10 mm in width, and 0.1 mm in thickness. The elastic modulus of the samples was determined by calculating the slope of the stress–strain curve at a 5% strain.

The dielectric properties of PU/NSiO_2_ were determined using an Agilent 4294A precision impedance analyzer (Agilent Technologies Co.) within a frequency range of 10^0^ Hz to 10^6^ Hz. The samples were prepared in a circular shape with a diameter of 7 mm. The breakdown strength measurements were processed by using a TREK610C instrument (PolyK Technologies) at a frequency of 100 Hz. The samples were prepared in the form of a circle with a diameter of 3 mm. The electric field-induced actuated thickness strains of PU/NSiO_2_ were determined using a piezo strain testing system with an MTI-2100 fiber optic probe. The samples were prepared in the form of a circle with a diameter of 7 mm. The sputtered gold was coated on both sides of the sample films as electrodes with a thickness of 30 nm.

## 3 Results and Discussion

### 3.1 Characterization of NSiO_2_

The FTIR spectra of SiO_2_ and NSiO_2_ are presented in Figure S1. It is evident that the spectra of SiO_2_ and NSiO_2_ are nearly the same. In comparison to SiO_2_, NSiO_2_ exhibits new peaks at 2970, 2927, and 2855 cm^–1^, which are ascribed to the stretching and bending vibrations of the –CH_2_ and –CH_3_ groups derived from APTES.^34^ However, the spectra of NSiO_2_ do not exhibit any discernible stretching vibration peaks of –NH_2_ at 3415 cm^–1^. This may be attributed to the overlap of the –NH_2_ peaks with the strong –OH peak. The morphology of SiO_2_ and NSiO_2_ is observed by SEM. As illustrated in Figure S2, both SiO_2_ and NSiO_2_ exhibit a uniform spherical structure, indicating that the modification has no discernible impact on the morphology of SiO_2_.

### 3.2 Microstructure of PU/NSiO_2_ Composites

**Figure 2:**
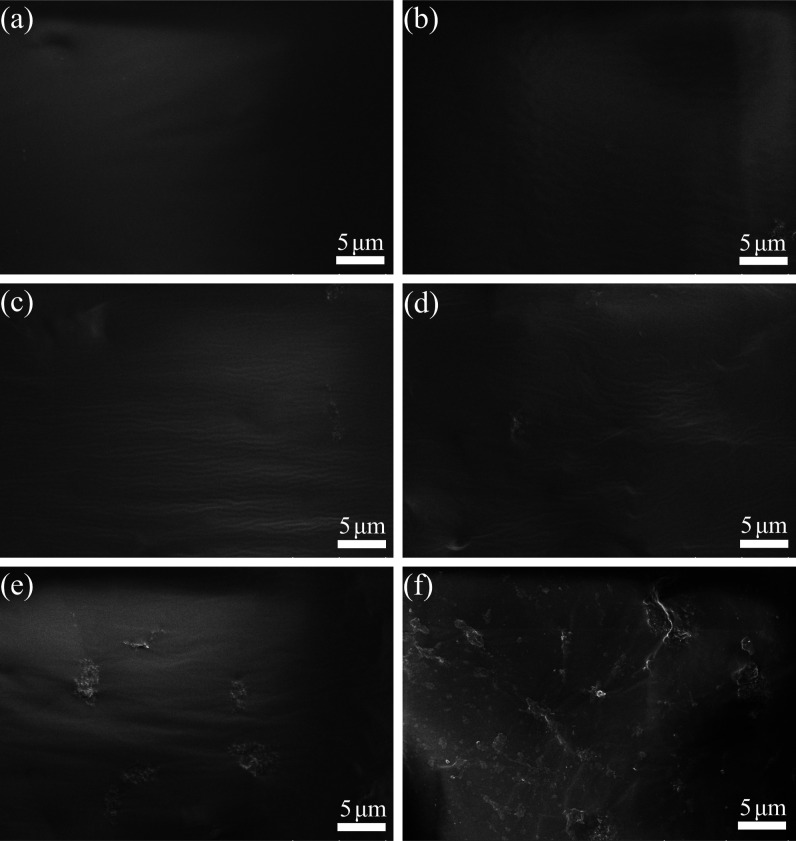
SEM images of (a) PU, (b) PU/NSiO_2_-1, (c) PU/NSiO_2_-2, (d) PU/NSiO_2_-3, (e) PU/NSiO_2_-4, and (f) PU/NSiO_2_-5.

The dispersion of SiO_2_ and NSiO_2_ in the PU could be observed through the morphology of the freeze-fractured composites. Figure S3 illustrates SEM images of the fracture section of the PU/SiO_2_-3 and PU/NSiO_2_-3 composites. The PU/SiO_2_-3 composite exhibits obvious agglomerated fillers. It can be observed that the PU/SiO_2_-3 composite exhibits an apparent phase separation. This can be attributed to poor dispersion and serious SiO_2_ aggregation. Conversely, NSiO_2_ appears to be distributed homogeneously within the PU matrix when the filler content is the same. NSiO_2_ is fully encapsulated within the PU matrix, indicating superior compatibility and wettability between NSiO_2_ and the PU matrix. Figure 2 illustrates the morphology of the freeze-fractured surfaces of the pure PU and PU/NSiO_2_ composites. As can be seen, pure PU exhibits smooth and flat fracture surfaces (Figure 2a). With increasing filler content, the fracture surfaces of samples (Figures 2b–d) display slight wrinkling. When the NSiO_2_ content exceeds 3%, the fracture surfaces of PU/NSiO_2_-4 and PU/NSiO_2_-5 become rough and display small areas of agglomeration and protrusion. This phenomenon may be attributed to the formation of filler agglomeration, as previously observed by other researchers.^35^

**Figure 3:**
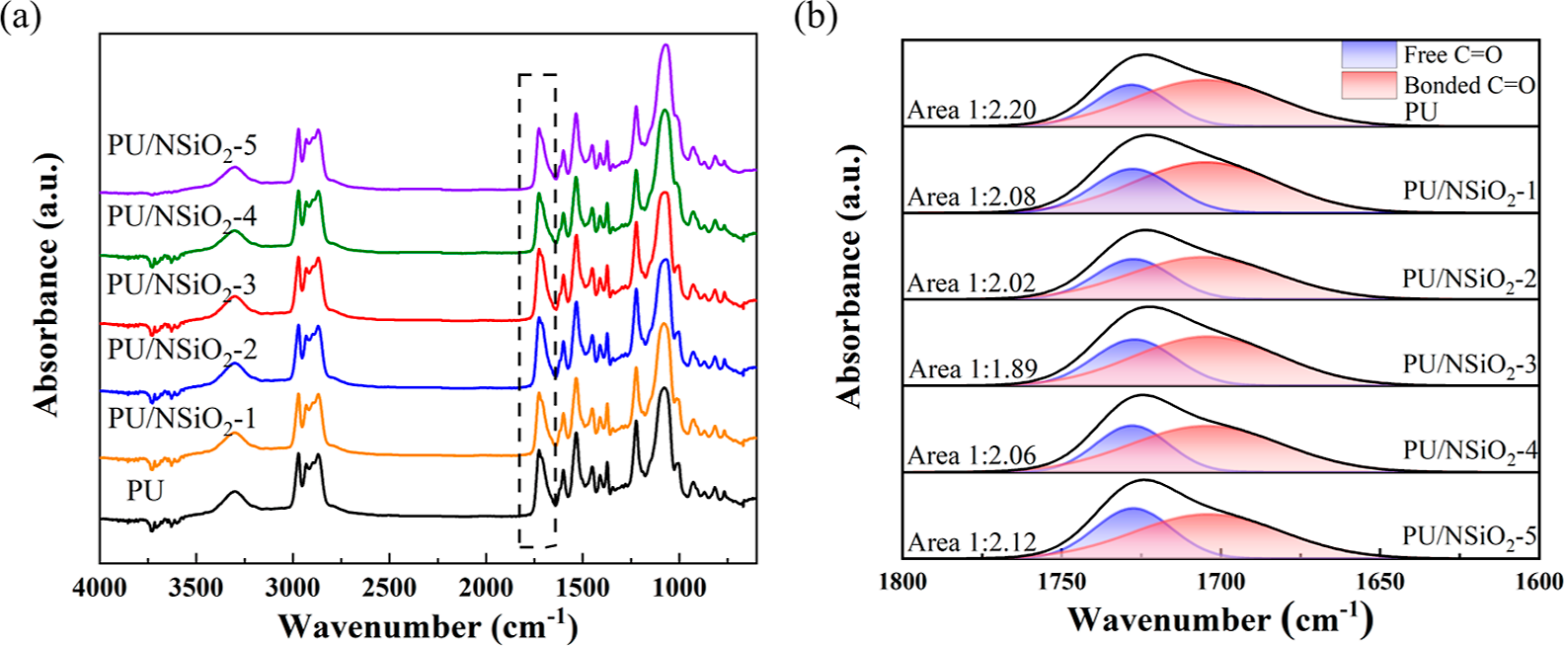
(a) FTIR spectra of PU and PU/NSiO_2_ composites and (b) curve fitting of the carbonyl stretching region of PU and PU/NSiO_2_ composites.

Infrared spectroscopy was employed to investigate the alteration in the hydrogen bonds in PU/NSiO_2_ composites. The infrared spectra of pure PU and the PU/NSiO_2_ composites are presented in Figure 3. It can be observed that the peak at approximately 3300 cm^–1^ is attributed to the N–H stretching vibration, while the peaks at 1560–1480 cm^–1^ are attributed to the amide II. The elastomer films do not display any absorption peaks corresponding to the stretching vibration of the –NCO at 2281 cm^–1^, indicating that the NCO groups derived from TDI are completely reacted to form urethane groups.^36^ The N–H groups of NSiO_2_ have the potential to disrupt the N–H/C=O hydrogen bonds of PU, thereby forming new hydrogen bonds with the bonded C=O groups in the HS of PU. The disruption of the N–H/C=O hydrogen bonds of PU results in the formation of free C=O groups of PU. Therefore, the addition of NSiO_2_ may result in a reduction in the intensity of the hydrogen-bonded C=O group of PU.

To gain further insight into the interaction between NSiO_2_ and PU, we conducted a detailed analysis of the change in the intensity of the peaks corresponding to the hydrogen-bonded C=O groups and the free C=O groups of PU using FTIR spectroscopy. Furthermore, a curve-fitting procedure based on a Gaussian distribution was employed to calculate the ratio of the peak area of the free C=O bands to that of the hydrogen-bonded C=O bands. The results are presented in Figure 3b. The peak at 1727 cm^–1^ represents the stretching vibration of free C=O groups of PU, whereas the peak at 1704 cm^–1^ represents the stretching vibration of hydrogen-bonded C=O groups of PU.^37^ The ratio of the peak area of free C=O bands to that of hydrogen-bonded C=O bands increased from 1:2.20 for pure PU to 1:1.89 for PU/NSiO_2_-3. This suggests that NSiO_2_ disrupts the N–H/C=O hydrogen bonds within the PU chains, thereby increasing the number of free C=O groups. As the filler content is increased further, the ratio of the peak area of free C=O bands to hydrogen-bonded C=O bands shows a slight decrease. This is because more of the NH_2_ groups of NSiO_2_ form hydrogen bonds with free C=O groups of PU, resulting in the increase in hydrogen-bonded C=O groups.

**Figure 4:**
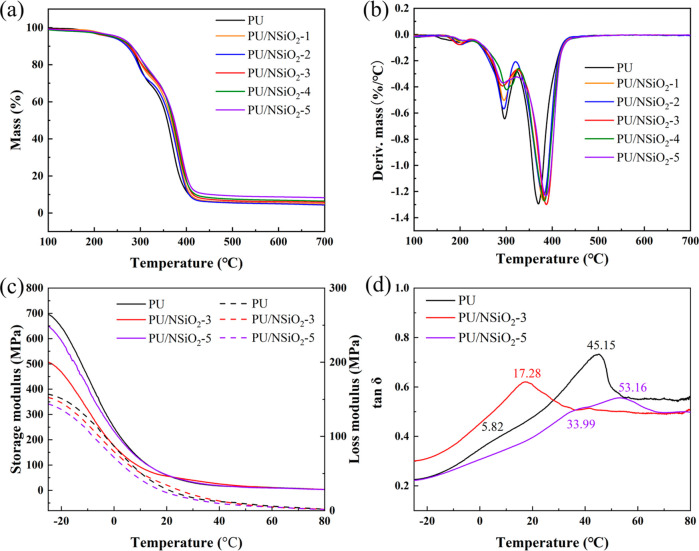
(a) TG and (b) DTG curves in nitrogen flow for PU and its composites. (c) Storage modulus and loss modulus–temperature curves and (d) tan δ–temperature curves of PU and its composites.

The thermal degradation and dynamic mechanical properties of PU/NSiO_2_ composites were investigated by TG and DMA. As illustrated in Figure 4a, the TG curves for PU and its composites exhibit a pronounced decline between 280 and 400 °C, until complete thermal degradation and carbonization at 600 °C. Two primary decomposition peaks in all samples are observed at approximately 296 and 370 °C, representing the maximum decomposition points of the hard segments and the soft segments, respectively. As the NSiO_2_ content increased, both degradation peaks were almost unchanged.

The storage modulus and loss modulus (Figure 4c) demonstrate a high value at a low temperature. As the temperature rises, the storage modulus and loss modulus of all samples drop sharply. Finally, the polymer network structure becomes unstable, resulting in the free motion of chain segments. The loss tangent (tan δ)–temperature curves of PU/NSiO_2_ composites are presented in Figure 4d. *T*_g_ represents the mobility level of polymer chains and networks in the matrix at the molecular level. It can be observed that PU exhibits two *T*_g_ peaks at 5.82 and 45.15 °C because of the presence of HS and SS. PU/NSiO_2_-3 exhibits a single *T*_g_ peak at 17.28 °C. This phenomenon may be attributed to the disruption of the HD of PU by NSiO_2_. The HD of PU breaks into multiple small hard domains and mixes with the SD to form a kind of nonideal two-phase system, so another *T*_g_ has not been observed in PU/NSiO_2_-3. However, PU/NSiO_2_-5 exhibits two *T*_g_ peaks at 33.99 and 53.16 °C again with the increment of NSiO_2_ contents, and the *T*_g_ values are higher than those of PU and PU/NSiO_2_-3. The increase of the *T*_g_ value indicates a decrease of molecular movement; it can be attributed to restrictions of chain movement by fillers. The tan δ values at *T*_g_ for both the PU/NSiO_2_-3 and PU/NSiO_2_-5 composites are lower than that of pure PU. The filler network is hard to destroy in the glass transition zone.^38^ Therefore, the energy dissipation of the matrix can primarily be attributed to the friction losses between the PU chains. With more filler added, the degree of confinement of the filler to the PU chains rises, thereby restricting the segmental relaxation of a proportion of the PU chains. Consequently, the tan δ values of the PU/NSiO_2_-3 and PU/NSiO_2_-5 composites exhibit a decline as more filler is added.

**Figure 5:**
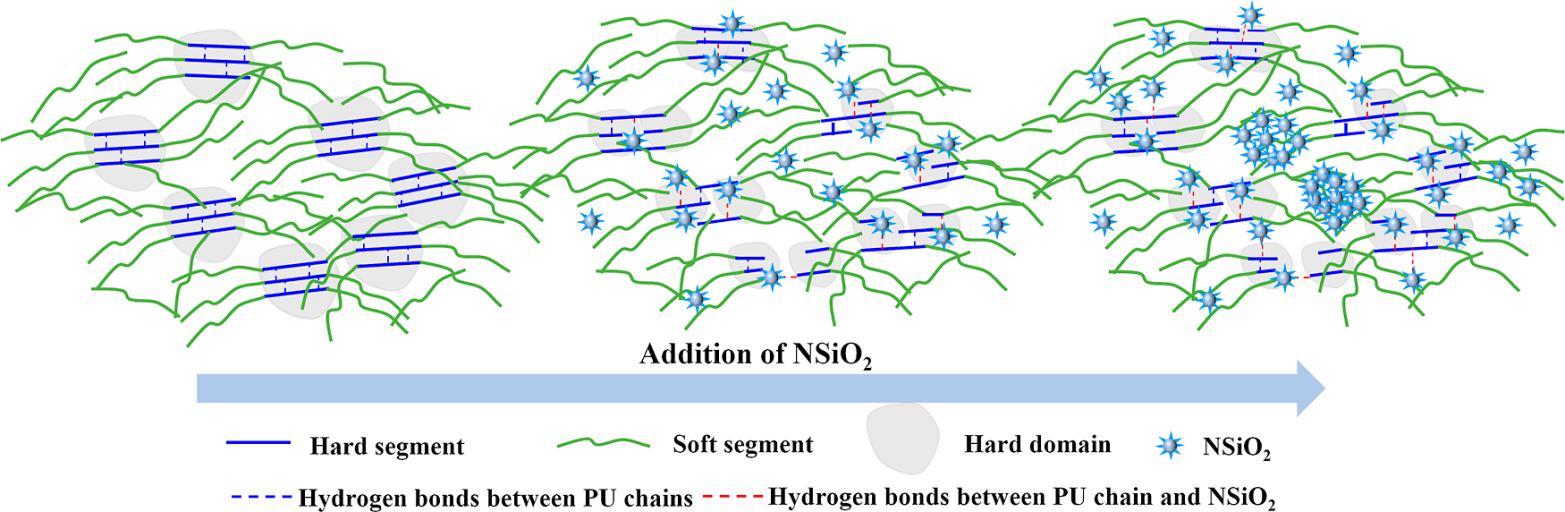
Schematic representation of the microstructure of PU with different contents of NSiO_2_.

In light of the aforementioned findings, the alterations in the hard domain structure and optimized hydrogen bonding structure of PU/NSiO_2_ composites are depicted in Figure 5.

### 3.3 Mechanical Properties of PU/NSiO_2_ Composites

**Figure 6:**
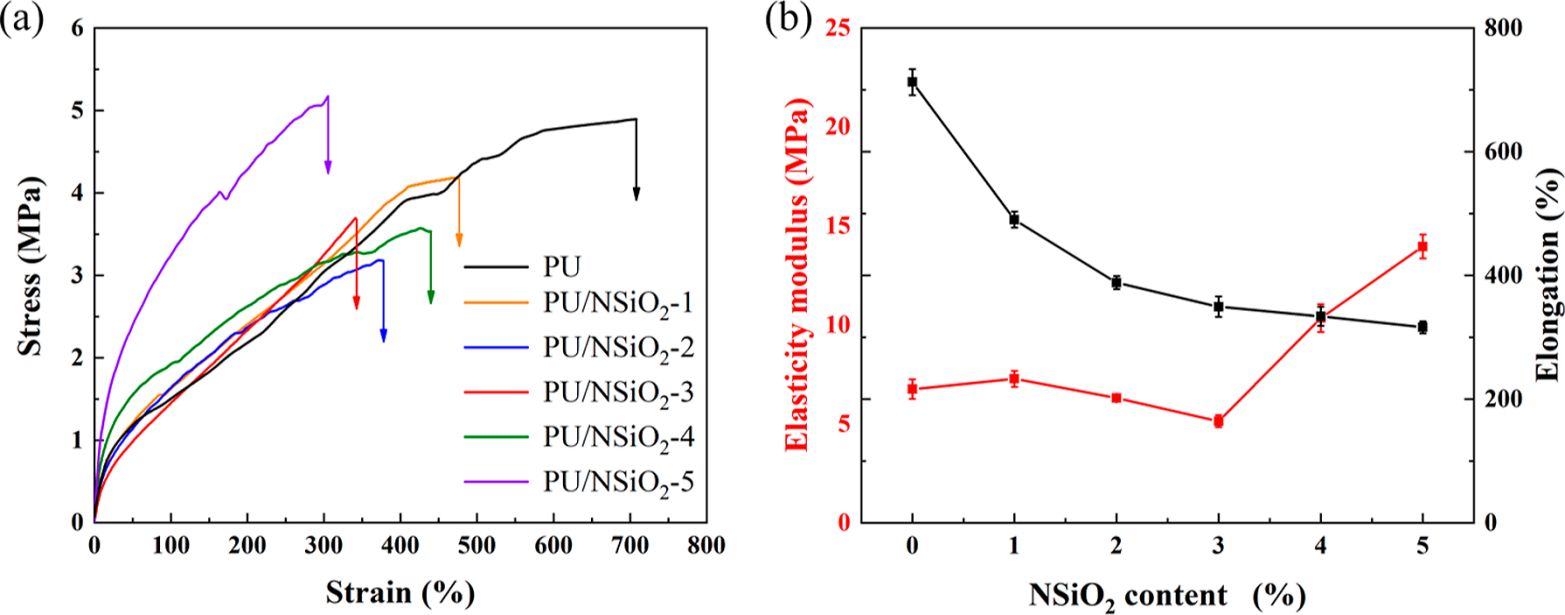
(a) Stress–strain curves of PU and PU/NSiO_2_ composites. (b) Elastic modulus (at 5% strain, red) and elongation (black) of PU and PU/NSiO_2_ composites.

The stress–strain curves of the PU/NSiO_2_ composites are presented in Figure 6. All composites demonstrate at least 300% strain. The data on the mechanical properties of all composites are presented in Table S3. The elastic modulus and tensile strength at break of PU are 6.80 ± 0.50 and 4.89 ± 0.11 MPa, respectively. When the NSiO_2_ content is 1%, the elastic modulus and tensile strength at break of PU/NSiO_2_-1 are 9.23 ± 0.50 MPa and 4.19 ± 0.09 MPa, respectively. However, the modulus, tensile strength, and elongation of PU/NSiO_2_ composites are not linearly dependent on the NSiO_2_ content. Among the PU/NSiO_2_ composites, PU/NSiO_2_-3 exhibits the lowest modulus and tensile strength. The elastic modulus of PU is primarily dependent on the HD of PU. The decrease of the modulus is mainly attributed to the disruption of the HD of PU by NSiO_2_. In addition, the elastic modulus and tensile strength of PU/NSiO_2_-5 are significantly higher than those of the other samples. This phenomenon may be attributed to the reinforcing effects of NSiO_2_, which restricts the mobility of polymer chains.^35,39^

### 3.4 Dielectric Properties of PU/NSiO_2_ Composites

**Figure 7:**
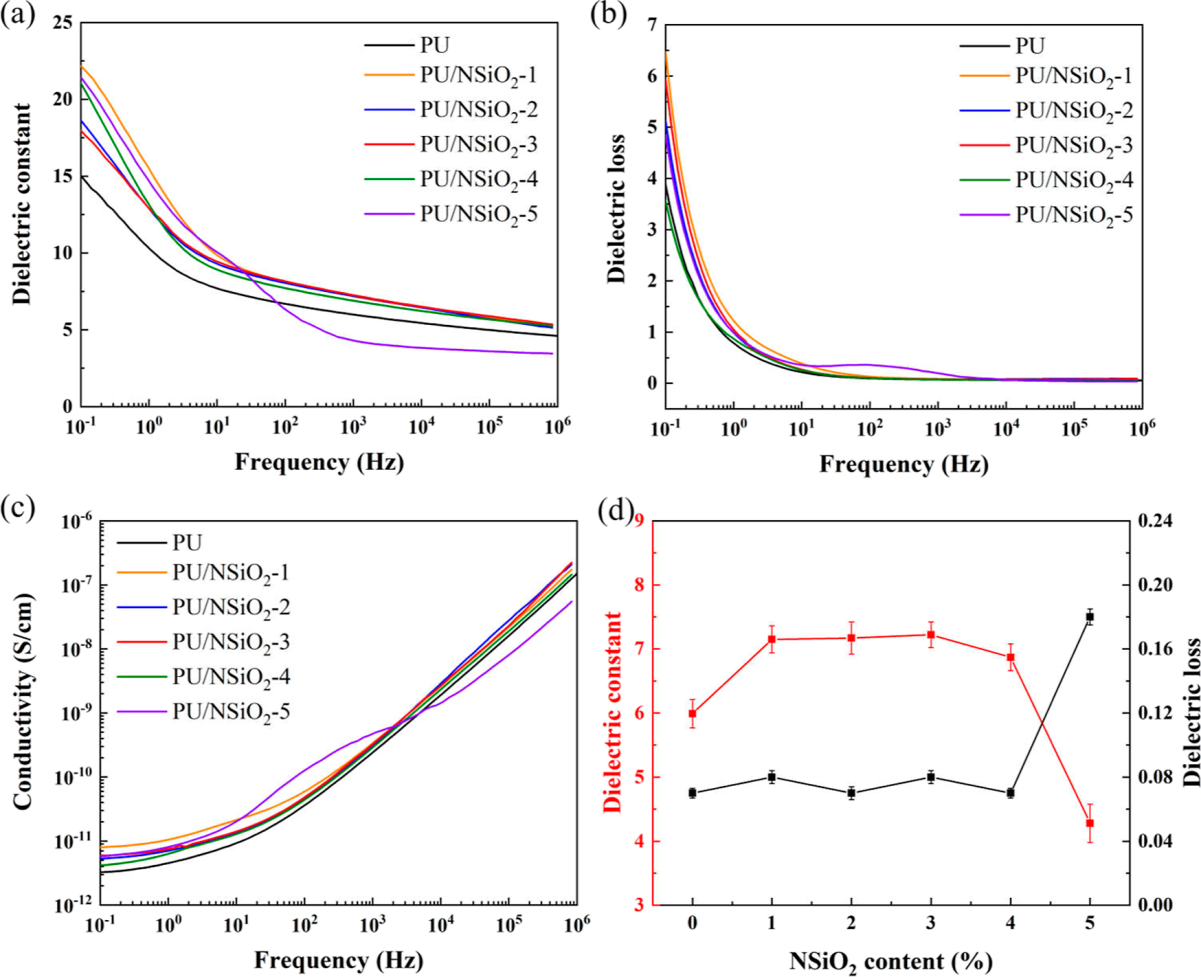
Frequency dependence of (a) dielectric constant, (b) dielectric loss, (c) conductivity, and (d) variations of dielectric constant and dielectric loss as a function of filler content at 10^3^ Hz of PU and PU/NSiO_2_ composites.

Figure 7 depicts the frequency-dependent dielectric properties of the PU/NSiO_2_ composites. As illustrated in Figure 7a, the dielectric constant of the composites demonstrates obvious frequency dependence, which decreases with the increase of frequency. This can be explained by the fact that the polarization of the sample is not able to keep up with the frequency of the electric field anymore.^40^ As illustrated in Table 1, ε_r_ at 10^3^ Hz obviously increases from 5.99 for pure PU to 7.22 for PU/NSiO_2_-3. The increase in ε_r_ by adding NSiO_2_ can be attributed to the disruption of the original N–H/C=O hydrogen bonds between PU chains, facilitating the dipole rotation of PU chain segments and consequently enhancing the polarizability of PU chains. It is noteworthy that ε_r_ decreases to 4.28 with the further increase in the content of NSiO_2_ to 5%. This is due to more of the NH_2_ groups of NSiO_2_ forming hydrogen bonds with free C=O groups of PU, limiting the mobility of the polarized groups and the dipole polarizability of PU. Moreover, the dielectric loss of PU and PU/NSiO_2_ composites also exhibits dependence on frequency, as illustrated in Figure 7b. The dielectric loss of all samples greatly decreases with the increase of frequency. The dielectric loss values at 10^3^ Hz for all samples are approximately 0.1. Figure 7c depicts the conductivity of all of the samples. The conductivity of all samples demonstrates a linear increase with the increase in frequency. In particular, the conductivity is less than 1 × 10^–11^ S/cm at low frequencies, which indicates the insulation characteristics of the PU/NSiO_2_ composites. However, under certain circumstances, the variation of the dielectric constant, dielectric loss, and conductivity of PU/NSiO_2_-5 with the frequency of the applied electric field differs significantly from that of other samples. It can be postulated that the complex is more susceptible to carrier migration than other samples. When this movement of charge carriers in the applied electric field occurs, the dielectric dissipation increases and the dielectric constant decreases.

**Table 1:** Electromechanical Properties of PU and PU/NSiO_2_ Composites

samples	dielectric content ε_r_ (10^3^ Hz)	dielectric loss (10^3^ Hz)	elastic modulus *Y* (MPa)	β = ε_r_/*Y* (MPa^–1^)
PU	5.99 ± 0.22	0.07 ± 0.003	6.76 ± 0.50	0.89
PU/NSiO_2_-1	7.15 ± 0.21	0.08 ± 0.004	7.28 ± 0.40	0.98
PU/NSiO_2_-2	7.17 ± 0.25	0.07 ± 0.004	6.31 ± 0.10	1.14
PU/NSiO_2_-3	7.22 ± 0.20	0.08 ± 0.004	5.14 ± 0.30	1.40
PU/NSiO_2_-4	6.87 ± 0.21	0.07 ± 0.003	10.35 ± 0.70	0.66
PU/NSiO_2_-5	4.28 ± 0.30	0.18 ± 0.005	13.96 ± 0.60	0.31

The incorporation of NSiO_2_ can simultaneously elevate the ε_r_ and reduce the *Y* of PU. Consequently, the electromechanical sensitivity (β = ε_r_/*Y*) of PU is observed to increase with the addition of NSiO_2_ (see Table 1). When the incorporation of NSiO_2_ is 3%, β of PU/NSiO_2_-3 increases to 1.40 MPa^–1^, which is an increase of 60% than that of PU (0.89 MPa^–1^).

### 3.5 Actuation Strain of PU/NSiO_2_ Composites

**Figure 8:**
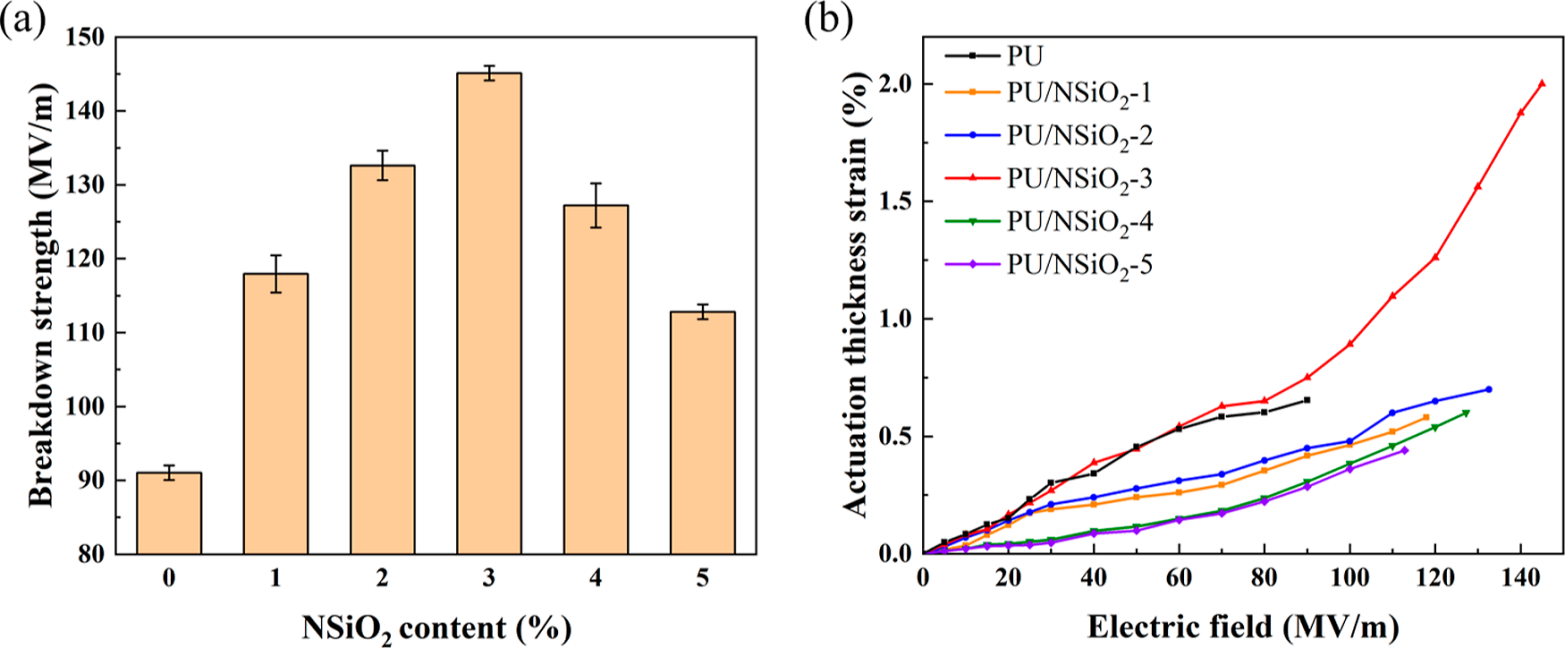
(a) Breakdown strength and (b) actuation thickness strain of PU and PU/NSiO_2_ composites.

Figure 8a shows the dependence of the breakdown strength on the NSiO_2_ content of PU. It can be observed that the breakdown strength of PU/NSiO_2_ composites is much higher than that of pure PU. The insulating nature of SiO_2_ results in a lower leakage current of PU/NSiO_2_ composites, which contributes to a higher electrical breakdown field. In addition, PU/NSiO_2_-3 exhibits the highest breakdown strength of 145.12MV/m, which is 1.6 times higher than that of pure PU (91.03MV/m). It should be noted that the higher electric breakdown field prevents the premature failure of DEA.^41^ Nevertheless, a further increase in filler content leads to a decrease in the breakdown strength of PU composites. This may be due to poor filler dispersion (more defects) and filler agglomeration. There are many space charges at the defects; these charges are considered to contribute to the leakage current through the influence of the electric field, resulting in lower electrical breakdown strength.^42^ This also corroborates the results in dielectric constant, dielectric loss, and conductivity.

Figure 8b shows the actuation thickness strain of PU/NSiO_2_ as a function of the electric field. The actuation thickness strain of the samples increases with increasing electric field. It can be easily observed that the maximum actuation strain of PU/NSiO_2_-3 is the largest, which is 2% at 145.12 MV/m. This can be attributed to increased ε_r_ and reduced *Y*, resulting in the largest β values. The electric field-induced maximum actuation strain of DEs is often limited by electrical breakdown. Thus, the maximum actuation strain of pure PU only stops at 0.65% due to the limitation of the low breakdown strength.

## 4 Conclusions

In this work, SiO_2_ was modified with APTES and incorporated into PU to prepare the PU/NSiO_2_ composites. The dielectric constant and electrical breakdown strength of PU/NSiO_2_-3 are simultaneously improved by introducing a suitable amount of NSiO_2_ into PU. In addition, the *Y* of PU is decreased due to the disruption of the HD of PU. As a result, the 60% increase in electromechanical sensitivity at 10^3^ Hz and the breakdown strength are achieved by the addition of 3% NSiO_2_. This study provides a simple and effective method for preparing DEs with improved dielectric performance for soft robotics and other applications related to future actuators and human–machine interfaces.

## Supporting Information Available

Mass of NSiO_2_ and the naming of corresponding samples; compounding formulation for PU/NSiO_2_ composites; FTIR spectra of SiO_2_ and NSiO_2_; SEM images of SiO_2_ and NSiO_2_; SEM images of PU/SiO_2_-3 and PU/NSiO_2_-3; and mechanical properties of PU and PU/NSiO_2_ composites (PDF)
