# Supplementary material for: Enhanced Mechanical and Dielectric Properties of Polyurethane Elastomers Containing Modified SiO2
Source: ACS Omega. 2024 Nov 15;9(47):47315–23. doi: 10.1021/acsomega.4c08565 (PMC11603205; doi:10.1021/acsomega.4c08565)
Supplement: Supplementary file 1 — ao4c08565_si_001.pdf [file ao4c08565_si_001.pdf]

Supporting information

Enhanced mechanical and dielectric properties of polyurethane elastomers containing modified SiO_2

*Miaomiao Qian, Xinru Wang, LiYang Yao, Yanchao Zhu **

College of Chemistry, Jilin University, Changchun, 130012, China

Corresponding author E-mail: yanchao_zhu@jlu.edu.cn

Table S1 Mass of filler and the naming of corresponding samples.

Samples	M_{filler} (g)
PU/$NSiO_2$ -1	0.11
PU/$NSiO_2$-2	0.23
PU/$NSiO_2$-3	0.34
PU/SiO_2-3	0.34
PU/$NSiO_2$-4	0.45
PU/$NSiO_2$-5	0.57

Table S2 Compounding formulation for PU/$NSiO_2$ composites

Ingredients	Mass (g)
PPG_{1000}	9
PPG_{2000}	2
TDI	4.36
$NSiO_2$	0.11-0.57
DMPA	0.94
DBTDL	3 drops
TEA	0.7

The FT-IR spectra of SiO_2 and $NSiO_2$ are shown in Figure S1. Obviously the spectrum of SiO_2 and $NSiO_2$ are almost the same：the peak at 3430 $cm^{-1}$is due to the stretching vibration of -OH. The peaks at 467 cm^{-1}, 799 cm^{-1} and 1093 cm^{-1} correspond to Si-O-Si swing, symmetric stretching and asymmetric stretching vibration, respectively. And the peak at 1630 cm^{-1} is attributed to the bending vibration of H_2O molecules. Compared with SiO_2, $NSiO_2$ shows new peaks at 2970 cm^{-1}、2927 cm^{-1} and 2855 cm^{-1} (indicated by the arrow) which are attributed to the

stretching and bending vibrations of the $-CH_2$ group $-CH_3$ group derived from APTES. However, there are no obvious stretching vibration peaks of $-NH_2$ at 3415 cm^{-1} appear in the spectra of $NSiO_2$. The reason may be that the peaks of $-NH_2$ are overlapped by the strong peak of -OH.

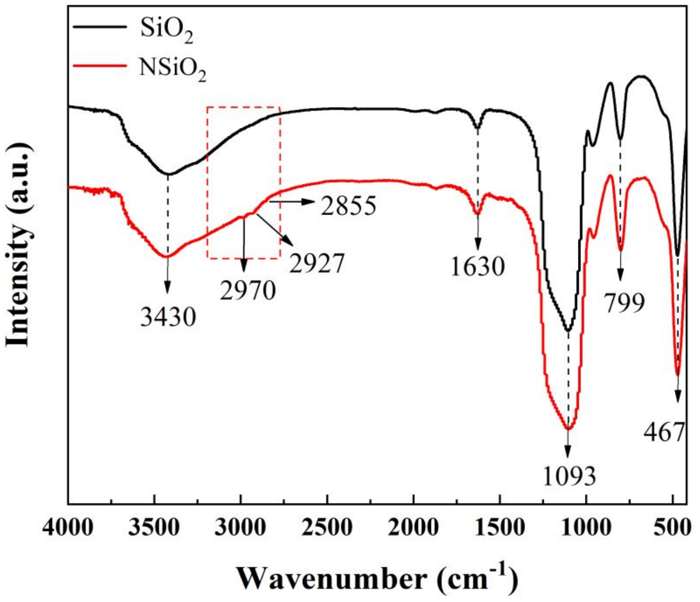

Figure S1. FT-IR spectra of SiO_2 and $NSiO_2$.

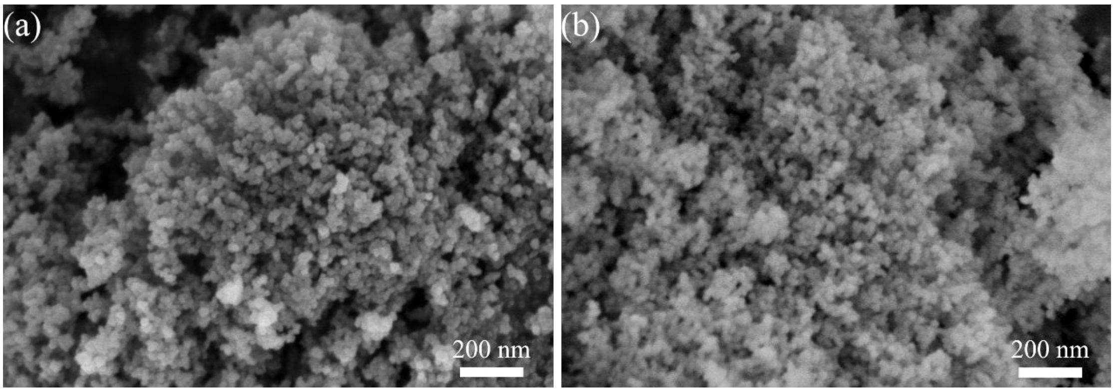

Figure S2. SEM images of (a) SiO_2 and (b) $NSiO_2$.

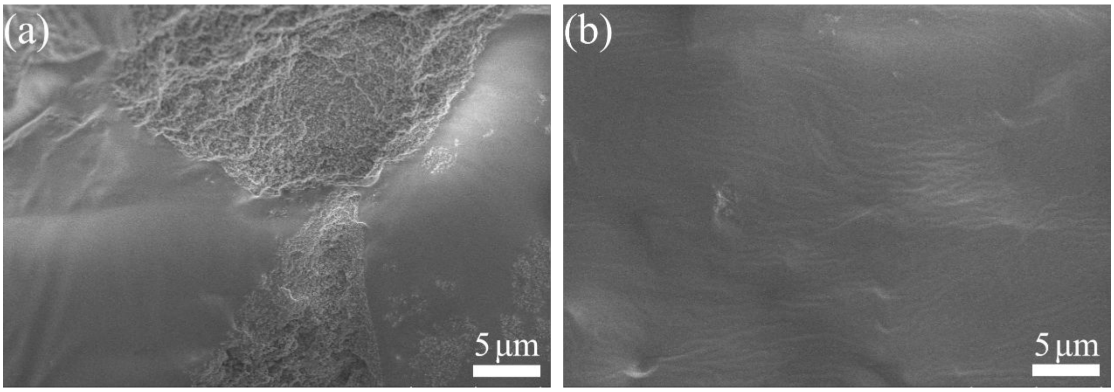

Figure S3. SEM images of (a) PU/ SiO_2-3 and (b) PU/ $NSiO_2$-3.

The mechanical properties of PU and PU/$NSiO_2$ composites are summarized in Table S3.

Table S3. Mechanical properties of PU and PU/$NSiO_2$ composites.

Samples	Tensile strength (MPa)	Elongation at break (%)
PU	4.89 ± 0.11	712.50 ± 21.03
PU/$NSiO_2$-1	4.19 ± 0.09	490.20 ± 12.95
PU/$NSiO_2$-2	3.18 ± 0.06	388.30 ± 10.66
PU/$NSiO_2$-3	3.69 ± 0.07	349.50 ± 16.51
PU/$NSiO_2$-4	3.53 ± 0.04	333.90 ± 15.22
PU/$NSiO_2$-5	5.17 ± 0.11	316.10 ± 9.86
